# High-fiber diet alters fecal microbiota and enhances fiber utilization and short-chain fatty acid production in tibetan pigs

**DOI:** 10.1186/s12866-026-05434-5

**Published:** 2026-07-27

**Authors:** Jiang Wang, Sichen Yang, Ying Yang, Chenguang Hu, Yucheng Wu, Feifan Zhang, Zhenda Shang, Peng Shang, Suozhu Liu, Kaifan Yu, Mengjie Zhang, Yan Lin, Zhonghua Cao, Zhankun Tan

**Affiliations:** 1 Xizang Plateau Feed Processing Engineering Research Center, Xizang Plateau Ecological Grassland Husbandry Collaborative Innovation Center, College of Animal Science, Xizang Agricultural and Animal Husbandry University, Linzhi, 860000 China; 2 National International Joint Research Center for Animal Gut Nutrition, Jiangsu Key Laboratory of Gastrointestinal Nutrition and Animal Health, Laboratory of Gastrointestinal Microbiology, College of Animal Science and Technology, Nanjing Agricultural University, Nanjing, 210095 China; 3 College of Engineering, China Agricultural University, Beijing , 100083 China

**Keywords:** Tibetan pig, High-fiber diet, Growth performance, Fiber degradation, Fecal microbiota

## Abstract

**Background:**

This study investigated the responses of growth performance, apparent nutrient digestibility, serum biochemical indices, fecal enzyme activity, short-chain fatty acids (SCFA), and fecal microbiota to a high-fiber diet in Tibetan pigs by manipulating dietary neutral detergent fiber (NDF) levels. Forty healthy Tibetan pigs with an initial body weight (IBW) of (13.46 ± 1.34) kg were randomly assigned to two groups: a control group (CON, NDF 8.80%) and a high-fiber group (HF, NDF 14.94%), with 20 replicates each and one pig per replicate. The experiment consisted of a 7-d adaptation period followed by a 28-d feeding trial.

**Results:**

Different dietary NDF levels had no significant effects on growth performance between the CON and HF groups. The digested amount of crude fiber (CF) was significantly increased in the HF group (*P* < 0.05). The HF group showed a significantly reduced apparent digestibility of acid detergent fiber (*P* < 0.05), while no significant differences were observed in the digestibility of other nutrients. Fecal cellulase activity was significantly higher in the HF group than in the CON group (*P* < 0.05). In addition, the concentrations of acetate, propionate, and butyrate in feces were significantly increased in the HF group (*P* < 0.05). The relative abundances of *Coprococcus* and *p-75-a5* were also significantly elevated (*P* < 0.05). Spearman correlation analysis revealed that *Coprococcus* abundance was positively correlated with isobutyrate levels, and *p-75-a5* was positively correlated with cellulase activity (*P* < 0.05).

**Conclusion:**

A high-fiber diet did not compromise growth performance in post-weaning Tibetan piglets and was associated with higher fecal cellulase activity, increased fecal acetate, propionate, and butyrate concentrations, and changes in selected bacterial taxa. These findings may provide useful reference information for the application of high-fiber diets in Tibetan pig production.

## Introduction

Tibetan pigs, an indigenous breed endemic to China, represent a vital genetic resource for biodiversity conservation due to their exceptional adaptability to the hypoxic and severely cold environments of the Qinghai-Tibetan Plateau [1]. Traditionally raised under free-range grazing systems, these pigs forage primarily on pasture grasses, plant roots, and fallen fruits, thereby evolving a remarkable tolerance to roughage [2]. While extensive research has elucidated the mechanisms underlying their adaptation to high altitudes [3–5] and hypoxia [6, 7], their capacity for digesting and utilizing neutral detergent fiber (NDF) remains poorly characterized and warrants systematic investigation. Evaluating their tolerance to dietary fiber (DF) could facilitate the incorporation of higher roughage levels in their diets, significantly alleviating feed costs. However, roughage tolerance is highly breed-dependent; for instance, while Mashen pigs can tolerate dietary NDF levels of up to 18%, the optimal NDF threshold for crossbred pigs (Duroc × Landrace × Yorkshire) is restricted to 13.5% [8]. Therefore, optimizing DF levels is of paramount importance to support the healthy production and economic viability of the Tibetan pig industry.

Agricultural by-products such as straw [9] and tubers [10] are widely used in feed production, resulting in diverse fiber compositions in diets. Fiber cannot be digested by enzymes secreted in the small intestine, but can be fermented by microorganisms residing in the hindgut [11]. Among them, bacteria such as *Prevotella* and *Fibrobacter*, which ferment carbohydrates and produce cellulase, are instrumental in this process [12]. DF level can affect the composition of the gut microbiota [13], indicating that diet is an important factor shaping microbial communities. This also suggests that the strong tolerance of Tibetan pigs to roughage may largely result from a greater adaptability of their gut microbiota to DF. In pigs fed high-fiber diets, the abundance of beneficial bacteria such as *Clostridia-UCG-014* and *Lactobacillus* increases significantly, while the abundance of *Streptococcus* decreases [14]. Fiber fermentation in the intestine produces short-chain fatty acids (SCFA), which serve as energy sources for intestinal cells, promote epithelial cell proliferation and intestinal development [15], and help improve the intestinal microbial environment by supporting beneficial bacteria [16]. After weaning, piglets often suffer from diarrhea, leading to high mortality and poor growth performance, greatly reducing production efficiency in pig farming [17]. Pathogenic diarrhea in piglets is mainly caused by *Escherichia coli* adhering to intestinal epithelial cells and releasing toxins [18]. However, increasing DF can induce changes in the gut microbiota and reduce diarrhea incidence in piglets [19]. Studies on the effects of NDF on the gut microbiota of healthy nursery Tibetan pigs remain limited. Although our previous study investigated the response of growing Tibetan pigs to high-fiber diets [20], it remains unclear how a high-fiber diet affects fiber digestion, fecal fermentation, and microbiota-related features in weaned Tibetan pigs during the nursery stage. Therefore, the present study assessed the effects of a high-fiber diet on growth performance, nutrient digestibility, blood parameters, fecal microbiota composition, fiber-degrading enzyme activity, and SCFA concentrations in weaned Tibetan pigs. In addition, associations among fecal microbiota, enzyme activity, and SCFA metabolism were analyzed to explore microbiota-related responses to dietary fiber. The findings of this study may provide stage-specific information for the application of dietary fiber in nursery Tibetan pig diets and offer a reference for diet formulation optimization.

## Materials and methods

This study was approved by the Animal Care and Use Committee of Xizang Agricultural and Animal Husbandry University (Approval No.: xza-2023-003).

### Animals, housing, experimental design, and diets

The experiment was conducted at the Animal Experimental Facility of Xizang Agricultural and Animal Husbandry University, Bayi District, Linzhi, Xizang Autonomous Region, China. The experimental pigs were all sourced from Tianhou Breeding and Planting Farmers and Herdsmen Professional Cooperative in Lang County, Linzhi City, Tibet Autonomous Region, China. A completely randomized design was adopted. Forty healthy weaned Tibetan pigs with similar genetic background and an average body weight (BW) of 13.46 ± 1.34 kg were selected and randomly assigned to two groups: a control group (CON; 8.80% NDF) and a high-fiber group (HF; 14.94% NDF), with 20 replicates per group and 1 pig per replicate. The experimental diets were formulated according to the Nutrient Requirements of Swine [21], and the composition and nutrient levels of the experimental diets (air-dry basis, %) are listed in Table 1. The wood cellulose used in the HF diet was a commercial insoluble structural fiber source derived from natural wood (mainly containing cellulose, hemicellulose, and lignin, with an acid detergent fiber content of approximately 70%, according to the manufacturer’s specification). Each pig was housed individually in a pen (1.5 × 2 m). A stainless-steel feeder was installed on the left side of the pen, and a nipple drinker was installed on the right side. Pigs were fed eight times per day, starting at 08:00, with a 2-h interval between feedings. After each feeding, only a small amount of feed remained in the feeder. During the trial, pigs had free access to feed and water. A 7-d adaptation period with a basal diet was conducted, followed by a 28-d experimental period.

**Table 1:** Composition and nutrient levels of the experimental diets (air-dry basis, %)

Item	CON	HF
Ingredients(%)		
Corn	33.33	28.89
Extruded corn	33.33	28.89
Soybean oil	-	2.95
Wood cellulose	-	4.90
Soybean meal	7.80	7.80
Extruded soybean	6.70	6.70
Fish meal	4.50	4.50
Soybean protein concentrate	3.00	4.10
Whey powder	7.00	7.00
Soybean hulls	1.50	1.50
CaCO_3_	0.70	0.73
CaHPO_4_	0.40	0.37
L-Lysine hydrochloride	0.23	0.20
DL-Methionine	0.04	0.05
DL-Threonine	0.11	0.10
Tryptophan	0.03	0.02
Salt	0.20	0.20
Choline	0.10	0.10
Premix^1^	0.23	0.23
Diatomite	0.80	0.80
Chemical Components^2^		
Digestible Energy (MJ/kg)	14.22	14.22
Crude Protein	16.50	16.50
Crude Fiber	3.00	6.00
Neutral Detergent Fiber	8.80	14.94
Acid Detergent Fiber	6.42	9.11
Calcium	0.70	0.70
Available Phosphorus	0.48	0.47
L-Lysine	1.10	1.10
Methionine + Cysteine	0.63	0.63
Threonine	0.74	0.74
Tryptophan	0.20	0.20
Acid-insoluble ash	0.80	0.80

*Abbreviations*: *CON*, control diet containing 8.80% NDF, *HF* high-fiber diet containing 14.94% NDF

1. Premix provided per kilogram of diet: Vitamin A 1700 IU, Vitamin D3 200 IU, Vitamin E 15 mg, Vitamin K3 0.5 mg, Thiamin 1.5 mg, Riboflavin 3 mg, Niacin 15 mg, Pantothenic acid 10 mg, Pyridoxine 1.5 mg, Folic acid 0.4 mg, Biotin 0.08 mg, Vitamin B12 15 mg, Fe 70 mg, Cu 4.5 mg, Mn 4 mg, Zn 75 mg, I 0.15 mg, Se 0.25 mg

2. Neutral detergent fiber (NDF) and acid detergent fiber (ADF) were measured values, while the other nutritional components were calculated

### Determination of growth performance

BW was recorded at weeks 0, 2, and 4 after 12 h of fasting with free access to water. The BW measured at the end of the adaptation period was considered the IBW during the experiment. To ensure accuracy, feed was withdrawn at 20:00 on the day before weighing, and weighing was conducted at 08:00 the following morning. During the trial, daily feed intake was recorded on a replicate basis. Average daily feed intake (ADFI), average daily gain (ADG), and feed-to-gain ratio (F/G) were calculated as follows.

$$\begin{aligned}\text{Average daily gain}\:\left(\mathrm{g/d}\right)=&\left(\text{Final body weight}-\text{Initial body weight}\right)\\&/\text{Number of experimental days}\end{aligned}$$

$$\begin{aligned}\text{Average daily feed intake}\left(\mathrm{g/d}\right)&=\left(\text{Total feed offered during the}\:\right.\\&\left.\text{ experimental period}-\text{Total feed}\;\right.\\&\left.\text{refused during the experimental period}\right)\\&/\text{Number of experimental days}\end{aligned}$$

$$\begin{aligned}\text{Feed-to-gain ratio}=&\text{Total feed intake}\:\text{during the}\\&\:\text{experimental period}\left(\mathrm{g}\right)\\&/\text{Total weight gain during}\\&\:\text{the experimental period}\left(\mathrm{g}\right)\end{aligned}$$

$$\begin{aligned}\text{Average daily fiber intake}\left(\mathrm{g/d}\right)&=\text{Average daily feed intake}\\&\times\text{Dietary fiber concentration}\end{aligned}$$

### Sample collection

On day 28 of the experimental period, blood samples were collected from all 10 pigs in each group via the anterior vena cava. Part of the blood was collected into tubes containing anticoagulant (EDTA-K2) for whole blood preparation, and the remaining blood was collected into vacuum tubes containing a procoagulant. The samples were centrifuged at 1800×g for 15 min (Centrifuge 5804, Eppendorf, Hamburg, Germany) to obtain serum. The serum was transferred into 1.5 mL centrifuge tubes and stored at − 20 °C until analysis.

From days 21 to 24 of the experimental period, fresh fecal samples were collected by rectal stimulation. Fresh feces from 10 pigs per group, avoiding contact with the ground, were collected using sterile cotton swabs and immediately placed into 1.5 mL sterile, enzyme-free cryogenic tubes. Aliquots of fresh fecal samples were immediately frozen in liquid nitrogen, transported to the laboratory, and stored at − 80 °C for 16 S rRNA sequencing, fiber-degrading enzyme activity assays, and SCFA analysis. The remaining fecal samples collected from each pig over the 4-day collection period were pooled, thoroughly homogenized, placed in sealed bags, and stored at − 20 °C for chemical analysis.

### Apparent nutrient digestibility analysis

Feed and collected fecal samples were dried, ground, and passed through a 1 mm sieve. All samples were analyzed in duplicate. If the difference between replicates exceeded 5%, the analysis was repeated. The proximate composition of feed and fecal samples was determined according to Association of Official Analytical Chemists (AOAC) [22] standard methods: crude protein was analyzed using method 984.13, crude fat using method 920.39, and crude fiber using method 978.10. NDF and acid detergent fiber (ADF) were determined according to the method of van Soest et al. [23] using a fiber analyzer (Ankom A2000i, NY, USA).

Acid-insoluble ash (AIA), which is indigestible and unabsorbed in the gastrointestinal tract, was used as an internal marker to calculate apparent nutrient digestibility. The apparent total tract digestibility (ATTD) was calculated as follows:$$\mathrm{ATTD}\left(\%\right)=\left[1-\left(\text{AIA content in feed/AIA content in feces}\right)\right.$$$$\left.\times\left(\text{Nutrient content in feces/Nutrient content in feed}\right)\right]\times100{\%}$$

$$\begin{aligned}\text{Total fiber digested}\left(\mathrm{g}\right)&=\text{Total feed intake}\\&\times\text{Dietary fiber concentration}\\&\times\text{Fiber digestibility}\end{aligned}$$

        

### Blood parameter analysis

Blood samples for hematological analysis were collected into tubes containing EDTA-K2 to prevent coagulation. After gentle inversion to ensure proper mixing, samples were analyzed immediately according to the operating procedures described in the user manual of the automatic hematology analyzer to avoid cell degradation.

All hematological parameters were measured using an automatic hematology analyzer (Dymind DH71CRP, Shenzhen, China). The parameters included white blood cells (WBC), red blood cells (RBC), hemoglobin (HGB), hematocrit (HCT), platelets (PLT), plateletcrit (PCT), mean corpuscular volume (MCV), mean corpuscular hemoglobin (MCH), mean corpuscular hemoglobin concentration (MCHC), mean platelet volume (MPV), platelet distribution width (PDW), and red cell distribution width (RDW).

All hematological analyses were completed within 2 h after sample collection to prevent artefacts and ensure data accuracy.

### Serum parameter analysis

Serum biochemical parameters were analyzed using an automatic biochemical analyzer (Toshiba BS120, Beijing, China) with commercial assay kits (Nanjing Jiancheng Bioengineering Institute, Nanjing, China). All procedures were performed according to the manufacturer’s instructions.

The measured serum biochemical indices included alanine aminotransferase (ALT), aspartate aminotransferase (AST), total protein (TP), albumin (ALB), globulin (GLO), alkaline phosphatase (ALP), urea (UREA), creatinine (CREA), total cholesterol (TC), and glucose (GLU). The AST/ALT ratio and albumin-to-globulin ratio (A/G) were calculated based on the measured values.

### Enzyme activity analysis

Cellulase and hemicellulase activities in fecal supernatants were determined. Fecal samples were homogenized in ice-cold saline at a ratio of 1:9 (g/mL). The homogenate was centrifuged at 2500 × g for 10 min at 4 °C, and the supernatant was collected for analysis.

Enzyme activities were measured using ELISA kits (Jiangsu Enzyme Immunoassay Co., Ltd., Nanjing, China) according to the manufacturer’s instructions. Blank wells, standard wells, and sample wells were prepared. For each standard well, 50 µL of standard solution was added. For each sample well, 40 µL of diluent and 10 µL of sample (fivefold diluted) were added. Samples were added to the bottom of the wells and gently mixed. The plate was sealed and incubated at 37 °C for 30 min, followed by five washes (30-fold diluted wash solution, 30 s each). Then, 50 µL of enzyme reagent was added to each well (except the blank), followed by incubation and washing. Subsequently, 50 µL of chromogenic solution A and 50 µL of chromogenic solution B were added and incubated at 37 °C in the dark for 10 min. The reaction was stopped by adding 50 µL of stop solution (color changed from blue to yellow). Optical density values were measured at 450 nm using a microplate reader (Thermo VARIOSKAN LUX, Shanghai Yituo Technology Co., Ltd., Shanghai, China) within 15 min, with the blank well as the reference.

### Fecal SCFA analysis

For each fecal sample, 0.2 g was mixed with 0.8 mL of 0.1 mol/L hydrochloric acid and vortexed thoroughly for 1 min to ensure complete homogenization, followed by incubation on ice for 30 min. The mixture was centrifuged at 13 000 ×g for 15 min using a Centrifuge 5804 (Eppendorf). The supernatant was filtered through a 0.22 μm membrane and analyzed for SCFA concentrations using a gas chromatograph (SCION 456i, Shanghai, China).

### Fecal microbial diversity analysis

Fecal samples were transported on dry ice to Shanghai Personal Biotechnology Co., Ltd. (Shanghai, China) for microbial diversity analysis. Total microbial DNA was extracted from the samples using the QIAamp Fast DNA Stool Mini Kit. The V3–V4 region of the bacterial 16 S rRNA gene was amplified using the forward primer F (5′-ACTCCTACGGGAGGCAGCA-3′) and reverse primer R (5′-GGACTACHVGGGTWTCTAAT-3′). Paired-end sequencing was performed on an Illumina MiSeq/NovaSeq platform using a PE250/PE300 sequencing strategy. Raw sequencing data were processed using QIIME2 software. Briefly, low-quality reads were filtered, paired-end reads were merged, and chimeric sequences were removed. The remaining high-quality sequences were clustered into operational taxonomic units (OTUs) at 97% sequence similarity using VSEARCH. Representative OTU sequences were aligned against the GenBank database to obtain taxonomic classification. Downstream analyses were performed using the OTU table at different taxonomic levels, including phylum, class, order, family, and genus. Microbial α-diversity was calculated using QIIME2 software, including Chao1, Faith’s_PD, Shannon, and Observed species indices, and the corresponding boxplots were generated using the ggplot2 package in R. Non-metric multidimensional scaling (NMDS) analysis was performed based on the Bray–Curtis distance matrix to evaluate differences in fecal microbial community structure between groups. Random forest analysis was performed at the genus level to identify potential marker taxa differentiating the CON and HF groups. The random forest model was trained with 100 trees, and model performance was evaluated using 10-fold cross-validation. Classification performance was assessed based on overall accuracy, the area under the receiver operating characteristic curve (AUC), sensitivity, specificity, and F1 score. The risk of overfitting was considered by comparing the cross-validated performance with the baseline accuracy. The top 15 genera were ranked according to feature importance and interpreted as exploratory marker taxa. A Venn diagram was generated to visualize the overlap between the top 20 most relatively abundant genera and the 15 key genera identified by the random forest model. Finally, Spearman’s rank correlation analysis (two-tailed test) was conducted using Origin 9.5.

### Statistical analysis

Raw data were organized using Microsoft Excel 2021. Normality and homogeneity of variance for continuous variables were tested using SPSS (version 27.0). The Shapiro–Wilk test was used to assess normality, and Levene’s test was used to evaluate homogeneity of variance. For variables that met the assumptions of normal distribution and homogeneity of variance, independent-samples t-tests were performed to compare differences between groups. Differences were considered significant at *P* < 0.05. All data are presented as mean ± standard error of the mean.

## Results

### Effects of a high-fiber diet on growth performance of tibetan pigs

No significant differences were observed in IBW between the two groups. Compared with the CON group, dietary NDF supplementation had no significant effects on the ADG, ADFI, or F/G of Tibetan piglets. However, the average daily intakes of both crude fiber (CF) and NDF were significantly higher in the HF group (*P* < 0.05; Table 2).

**Table 2:** Effects of a high-fiber diet on growth performance of Tibetan pigs

Item	CON	HF	SEM	*P*-value
1–2 weeks				
IBW (kg)	13.25	13.68	0.299	0.486
FBW (kg)	17.75	17.93	0.342	0.799
ADG (g/d)	321.55	303.76	14.439	0.552
ADFI (g/d)	962.99	905.32	27.932	0.315
F:G	3.04	3.07	0.103	0.896
3–4 weeks				
FBW (kg)	23.37	23.14	0.325	0.731
ADG (g/d)	401.81	372.40	11.431	0.207
ADFI (g/d)	1205.60	1176.33	32.500	0.665
F:G	3.00	3.19	0.072	0.209
1–4 weeks				
ADG (g/d)	361.67	338.08	8.767	0.192
ADFI (g/d)	1084.30	1040.83	22.911	0.357
F:G	3.00	3.11	0.073	0.456
Average daily fiber intake (g/d)				
CF	32.53	62.45	3.592	<0.001
ADF	69.61	94.82	3.386	<0.001
NDF	95.41	155.50	7.416	<0.001

*Abbreviation*: *CON* control diet containing 8.80% NDF, *HF* high-fiber diet containing 14.94% NDF, *IBW* initial body weight, *FBW* final body weight, *ADG* average daily gain, *ADFI* average daily feed intake, *F/G* feed-to-gain ratio. *P* < 0.05 indicates a significant difference, *P* > 0.05 indicates no significant difference. The same applies to the tables below

### Effects of a high-fiber diet on apparent digestibility in tibetan pigs

No significant differences were observed in the apparent digestibility of CP, EE, CF, or NDF, nor in the digestible intake of ADF and NDF between the HF and CON groups. However, compared with the CON group, the apparent digestibility of ADF was significantly reduced in the HF group (*P* < 0.05). Furthermore, the amount of digested CF was significantly higher in the HF group than in the CON group (*P* = 0.002; Table 3).

**Table 3:** Effects of a high-fiber diet on apparent nutrient digestibility and digested fiber amount in Tibetan pigs

Item	CON	HF	SEM	*P*-value
Apparent Digestibility				
CP (%)	76.54	76.05	0.735	0.753
EE (%)	71.25	68.82	1.165	0.314
CF (%)	42.23	44.25	1.150	0.521
ADF (%)	23.17	18.15	1.171	0.026
NDF (%)	36.12	34.32	0.794	0.272
Total fiber digested				
CF (g)	345.62	698.99	62.947	0.002
ADF (g)	402.94	390.03	44.138	0.872
NDF (g)	860.33	1205.65	128.356	0.137

*Abbreviation*: *CON* control diet containing 8.80% NDF, *HF* high-fiber diet containing 14.94% NDF, *CP* crude protein, *EE* ether extract, *CF* crude fiber, *ADF* acid detergent fiber, *NDF* neutral detergent fiber

### Effects of a high-fiber diet on blood parameters of tibetan pigs

No significant differences were noted between the HF and CON groups in WBC, RBC, HGB, HCT, MCV, MCH, MCHC, RDW, PLT, MPV, PDW, or PCT levels (Table 4).

**Table 4:** Effects of a high-fiber diet on blood parameters of Tibetan pigs

Item	CON	HF	SEM	*P*-value
WBC (10^9^/L)	22.86	21.94	0.716	0.535
RBC (10^12^/L)	8.59	8.41	0.131	0.520
HGB (g/L)	144.40	145.80	2.981	0.822
HCT (%)	45.77	45.48	0.948	0.883
MCV (fL)	53.29	54.15	0.700	0.554
MCH (pg)	16.75	17.29	0.258	0.306
MCHC (g/L)	320.20	320.00	1.053	0.927
RDW (%)	18.23	18.22	0.320	0.988
PLT (10^9^/L)	442.50	389.30	25.311	0.306
MPV (fL)	6.78	7.04	0.078	0.095
PDW (%)	16.02	16.34	0.084	0.054
PCT (%)	0.29	0.27	0.017	0.438

*Abbreviation*:*CON* control diet containing 8.80% NDF, *HF* high-fiber diet containing 14.94% NDF, *WBC* white blood cells, *RBC* red blood cells, *HGB* hemoglobin, *HCT* hematocrit, *MCV* mean red blood cell volume, *MCH* mean corpuscular hemoglobin content, *MCHC* mean corpuscular hemoglobin concentration, *RDW* red blood cell distribution width, *PLT* platelets, *MPV* mean platelet volume, *PDW* platelet distribution width, *PCT* plateletcrit

### Effects of a high-fiber diet on serum parameters of tibetan pigs

No significant differences were observed between the HF and CON groups in ALT, AST, AST/ALT, ALP, TP, ALB, GLO, A/G, UREA, CREA, TC, or GLU levels (Table 5).

**Table 5:** Effects of a high-fiber diet on serum biochemical indices of Tibetan pigs

Item	CON	HF	SEM	*P*-value
ALT (U/L)	73.49	67.51	3.028	0.346
AST (U/L)	39.68	40.38	2.905	0.907
AST/ALT	0.56	0.58	0.037	0.701
ALP (U/L)	174.40	158.20	6.964	0.255
TP (g/L)	75.78	72.54	1.218	0.196
ALB (g/L)	44.66	43.53	0.586	0.353
GLO (g/L)	31.07	33.50	1.226	0.343
A/G	1.41	1.34	0.035	0.375
UREA (mmol/L)	6.71	6.44	0.144	0.364
CREA (µmol/L)	73.85	68.97	2.028	0.239
TC (mmol/L)	3.30	3.17	0.087	0.482
GLU (mmol/L)	5.11	5.14	0.150	0.934

*Abbreviation*: *CON* control diet containing 8.80% NDF, *HF* high-fiber diet containing 14.94% NDF, *ALT* alanine aminotransferase, *AST* aspartate aminotransferase, *AST/ALT* aspartate aminotransferase to alanine aminotransferase ratio, *AST/ALT* was calculated for each individual animal before group means were obtained. *TP* total protein, *ALB* albumin, *GLO* globulin, *A/G* albumin/globulin ratio, *UREA* urea, *CREA* creatinine, *TC* total cholesterol, *GLU* glucose measurement

### Effects of a high-fiber diet on fecal enzyme activity and scfa concentrations in tibetan pigs

**Fig. 1:**
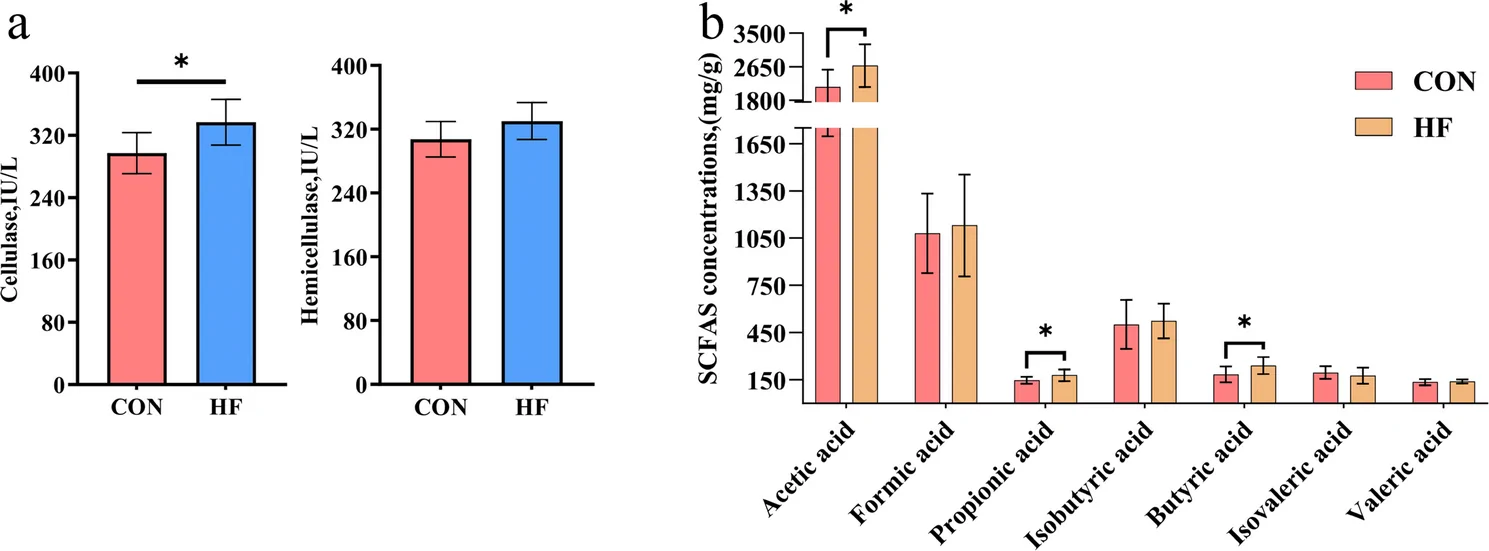
Effects of a high-fiber diet on fecal enzyme activity and short-chain fatty acid concentrations in nursery Tibetan pigs. CON, control diet containing 8.80% NDF; HF, high-fiber diet containing 14.94% NDF. **a** Cellulase and hemicellulase activities; **b** SCFA concentrations. “*” *P* < 0.05

Cellulase activity in the HF group was significantly higher than that in the CON group (*P* < 0.05), while no significant difference in hemicellulase activity was observed between the two groups. The concentrations of acetate, propionate, and butyrate in the HF group were significantly higher than those in the CON group (*P* < 0.05). However, no significant differences were observed between the CON and HF groups in the concentrations of formate, isobutyrate, valerate, or isovalerate (Fig. 1).

### Effects of a high-fiber diet on fecal microbiota in tibetan pigs

**Fig. 2:**
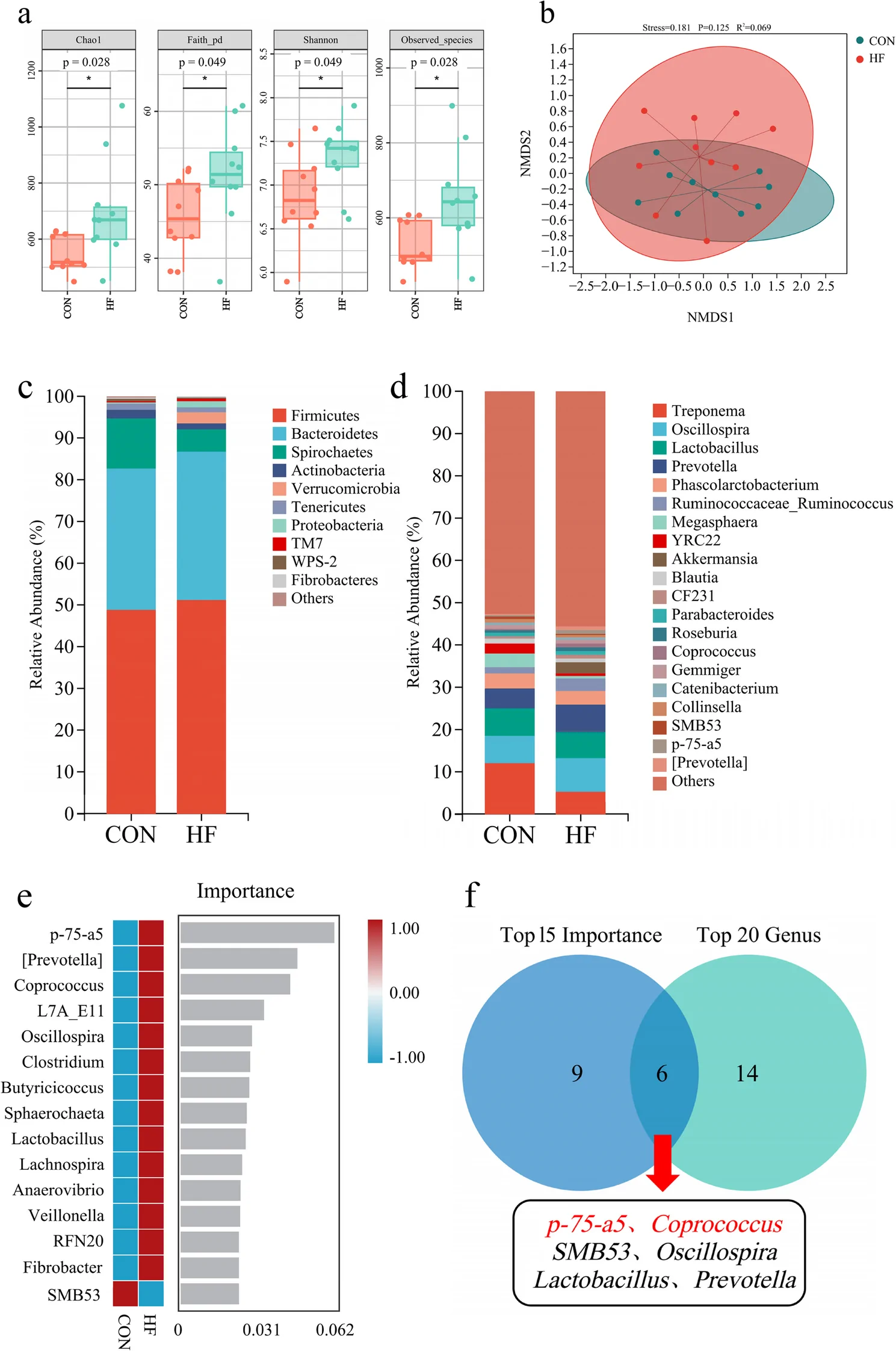
Effects of dietary fiber level on fecal microbial communities in Tibetan pigs. CON, control diet containing 8.80% NDF; HF, high-fiber diet containing 14.94% NDF. **a** α-diversity indices among groups.“*” *P* < 0.05. **b** Non-metric multidimensional scaling (NMDS) plot between groups based on Bray-Curtis dissimilarity (stress = 0.1810). Statistical analysis via PERMANOVA confirmed no significant difference in microbial community structure between the CON and HF groups (R2 = 0.069, *P* = 0.125, permutations = 999), and the homogeneity of multivariate dispersions was verified via the PERMDISP test (F = 0.189, *P*= 0.638). **c** Top 10 phyla based on relative abundance at the phylum level. **d** Top 20 genera based on relative abundance at the genus level, “Others” represents the combined relative abundance of genera outside the top 20. **e** Top 15 important genera identified by random forest analysis at the genus level 100 trees; 10-fold cross-validation; overall accuracy = 0.55, AUC = 0.55, sensitivity = 0.30, specificity = 0.80, F1 score = 0.40; baseline accuracy = 0.50). These genera were interpreted as exploratory marker taxa rather than definitive biomarkers. **f** Venn diagram showing six overlapping genera between important taxa and top abundant taxa

The Chao1, Faith’s_PD, Observed species, and Shannon indices were significantly higher in the HF group than in the CON group (*P* < 0.05; Fig. 2a). NMDS was used to analyze β-diversity of the microbiota. The stress value was 0.181, indicating reliable ordination. PERMANOVA showed no significant difference in microbial community structure between the CON and HF groups (R² = 0.069, *P* = 0.125, 999 permutations), and PERMDISP confirmed homogeneous dispersion between groups (F = 0.189, *P* = 0.638;Figure 2b).

At the phylum level, the dominant phyla in both groups were Firmicutes, Bacteroidetes, and Spirochaetes, accounting for 90% of the total abundance (Fig. 2c). At the genus level, the top 20 genera in both groups are shown in Fig. 2d. Within Firmicutes, the dominant genera included *Oscillospira*, *Lactobacillus*, *Phascolarctobacterium*, *Ruminococcaceae_Ruminococcus*, and *Megasphaera*; within Spirochaetes, the dominant genus was *Treponema*; within Bacteroidetes, the dominant genus was *Prevotella*.

The top 15 important genera identified by the random forest model are shown in Fig. 2e. Figure 2f presents a Venn diagram showing the overlap between the top 20 genera based on relative abundance and top 15 important genera identified by random forest analysis at the genus level in the CON and HF groups. The overlapping genera were *p-75-a5*, *SMB53*, *Coprococcus*, *Oscillospira*, *Lactobacillus*, and *Prevotella*.

**Fig. 3:**
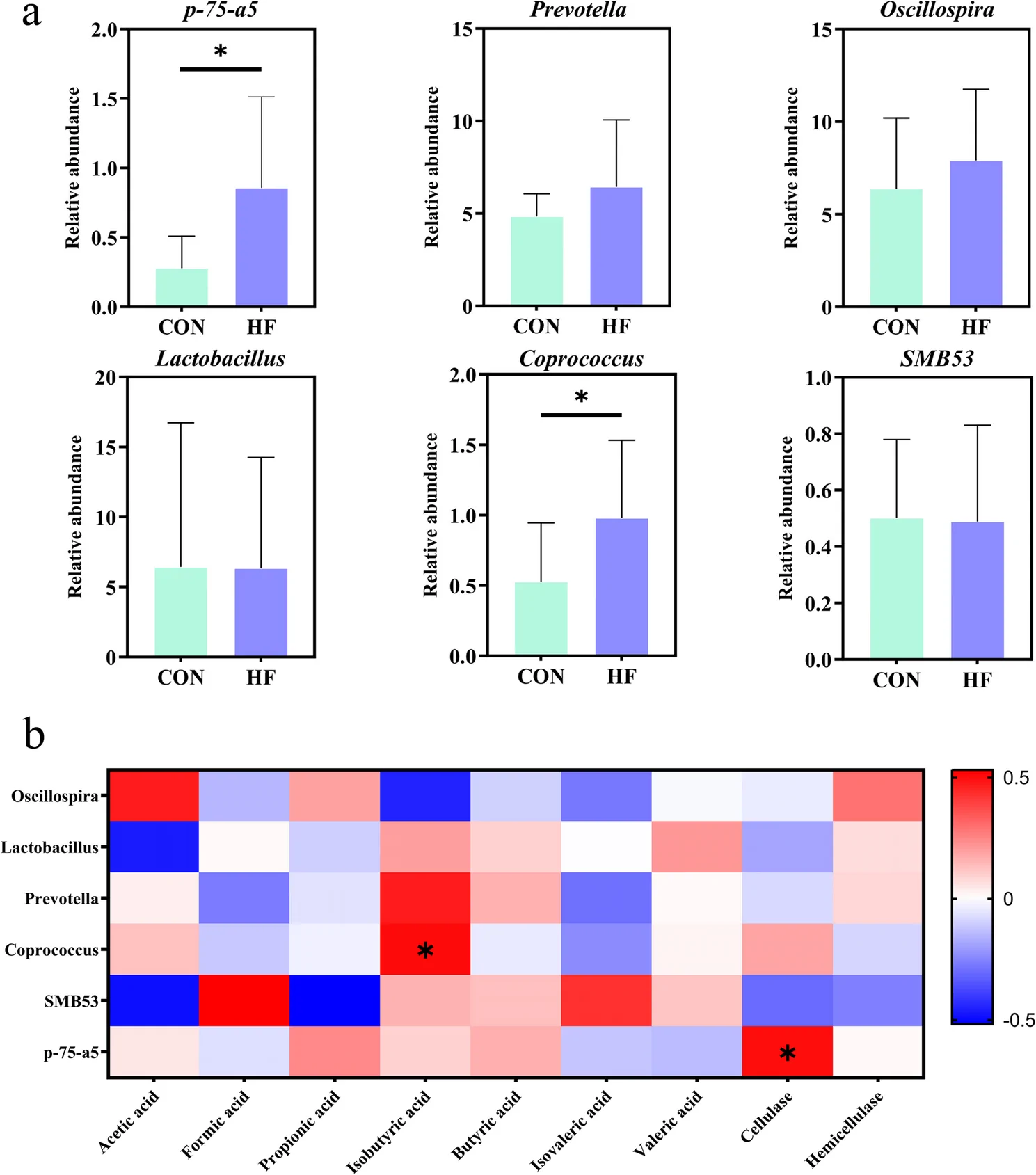
Effects of dietary fiber level on the relative abundance of fecal microbiota in Tibetan pigs. CON, control diet containing 8.80% NDF; HF, high-fiber diet containing 14.94% NDF. **a** Differences in the relative abundance of marker genera between the two groups. “*” *P* < 0.05. **b** Spearman correlation analysis between marker genera and fiber degradation indices. Red indicates positive correlation; blue indicates negative correlation; “*” indicates significant correlation at *P* < 0.05

Figure 3a shows differences in the relative abundance of marker genera between the two groups. The relative abundances of *Coprococcus* and *p-75-a5* were significantly higher in the HF group than in the CON group (*P* < 0.05; Fig. 3a). No significant differences were observed in the relative abundances of *Oscillospira*, *Lactobacillus*, *Prevotella*, or *SMB53* between the two treatments. As shown in Fig. 3b, *Coprococcus* was positively correlated with isobutyrate concentration, and *p-75-a5* was positively correlated with cellulase activity.

## Discussion

### Effects of a high-fiber diet on growth performance of tibetan pigs

Current evidence indicates that dietary fiber contributes to pig growth and development, specifically by enhancing gastrointestinal function and regulating intestinal motility [24]. However, the inclusion of fiber in swine diets is often restricted because excessive levels can negatively impact energy and nutrient digestibility [25, 26]. Furthermore, the effects of dietary fiber are highly dependent on its type and source [27], as well as the breed and growth stage of the pig [28, 29]. For instance, Li et al. reported that adding 4.95% crude fiber to the diet of growing pigs (Duroc × Landrace × Yorkshire) had no adverse effects on growth performance [30]. Conversely, Hu et al. found that a diet containing 7% crude fiber significantly reduced the chest circumference and ADFI of finishing pigs (Landrace × Large White × Duroc) [31]. Previous studies have also demonstrated that Tibetan pigs can adapt to high-fiber diets (10% crude fiber) without compromising their growth performance [20]. Thus, breed and growth stage are key determinants of fiber tolerance. In the present study, a diet containing 14.94% NDF did not negatively affect the ADFI, ADG, or F/G of Tibetan pigs, suggesting that this fiber level did not exceed their tolerance under the present experimental conditions. As an indigenous breed adapted to the high-altitude plateau environment, Tibetan pigs possess a unique digestive tract structure and microbial ecosystem that confer strong fiber tolerance and utilization capacity [32]. Providing an appropriate fiber level may help maintain intestinal homeostasis, support hindgut fermentation, and preserve gut barrier function, presumably contributing to stable production performance. Because the additional fiber in the HF diet was mainly supplied by wood cellulose, a relatively purified source of insoluble structural fiber, the present findings should be interpreted as the response of Tibetan pigs to increased insoluble structural fiber rather than to the full complexity of natural roughage. Nevertheless, because such structural fiber is closely related to the NDF and ADF fractions of common roughage sources [33], these results may provide a controlled reference for evaluating fiber utilization in Tibetan pigs. Future studies comparing purified and natural fiber sources are needed to clarify the effects of fiber source and physicochemical properties on fiber utilization and gut microbiota responses.

### Effects of a high-fiber diet on nutrient utilization in tibetan pigs

The nutritional and physiological effects of dietary fiber depend largely on its composition. For instance, soluble dietary fiber (SDF) and insoluble dietary fiber (IDF) exert distinct physiological roles. IDF reduces pathogen proliferation by shortening digesta transit time [34], whereas SDF is considered a preferred substrate for the microbial production of SCFA. These SCFA play crucial roles in host glucose and lipid metabolism, as well as intestinal health [35]. In the present study, the high-fiber diet had no significant effects on the digestibility of CP, EE, CF, or NDF, but it significantly reduced ADF digestibility. This decrease may be related to the increased proportion of purified, insoluble, and relatively crystalline cellulose in the HF diet, which may be less accessible to microbial fermentation than mixed natural roughage containing hemicellulose, pectin, and soluble fiber fractions [36, 37]. Notably, the total digested amounts of CF and NDF in the HF group exceeded those in the CON group, with the increase in CF being statistically significant. This result should be interpreted primarily as a consequence of increased dietary fiber intake rather than enhanced proportional fiber digestibility. In the present study, the high-fiber diet increased the total amount of digested crude fiber in Tibetan pigs. This finding is consistent with the significantly elevated fecal cellulase activity and increased SCFA concentrations observed in the HF group, suggesting that the increased amount of digested fiber was accompanied by changes in fermentation-related indicators, which could partially contribute to host energy supply [38]. Furthermore, our results are consistent with those of Luo et al., who reported that supplementing piglet diets with 8% unfermented wheat bran significantly reduced ADF and NDF digestibility while increasing crude fiber digestibility [39]. The observed reduction in specific fiber digestibility did not negatively impact growth performance, which may be explained by the additional energy supplied through enhanced hindgut microbial fermentation of the increased dietary fiber [40]. Conversely, other studies have found that adding 7% crude fiber to finishing pig diets reduced overall fiber digestibility (CF, NDF, ADF) and compromised growth performance [31]. These discrepancies across studies are likely attributable to variations in fiber sources, inclusion levels, and experimental conditions.

### Effects of a high-fiber diet on blood parameters in tibetan pigs

The hematological and biochemical profiles of Tibetan pigs at high altitude are closely related to their adaptation to hypoxia and disease resistance [41]. Moreover, serum biochemical parameters serve as vital indicators of nutrient utilization, overall health status, and growth performance [42]. In the present study, hematological and serum parameters were measured to assess the metabolic impact of the high-fiber diet. Our results indicated no adverse effects on any of the measured blood indices. These findings are consistent with Lamu et al. [20], who reported that a high-fiber diet successfully maintained overall health and basal metabolism in Tibetan pigs.These findings suggest that dietary fiber within a tolerable range may not cause detectable adverse changes in hematological or serum biochemical parameters in pigs [43].

### Effects of a high-fiber diet on fecal enzyme activity and SCFA

Endogenous digestive enzymes in pigs have limited capacity to degrade dietary fiber; instead, dietary fiber is mainly fermented by hindgut microbiota with the involvement of microbial enzymes [44]. Therefore, fecal fiber-degrading enzyme activity can, to some extent, reflect microbial fiber fermentation capacity and is closely related to intestinal health [45–47]. In the present study, fecal cellulase activity was significantly higher in the HF group, which is consistent with Lamu et al. [20], who reported increased intestinal cellulase and hemicellulase activities in Tibetan pigs fed high-fiber diets. Increased dietary fiber may provide substrates for fiber-degrading bacteria and promote microbial fermentation, leading to the production of SCFA, including acetate, propionate, and butyrate [48–49]. These metabolites serve as important energy substrates and are involved in intestinal epithelial development [50–52], nutrient absorption [53], and barrier-related functions [54]. Previous work has suggested that microbial populations involved in propionate and butyrate metabolism are enriched in Tibetan pigs, which may be related to energy metabolism and adaptation to plateau environments [55]. In the present study, the increases in fecal cellulase activity and fecal acetate, propionate, and butyrate concentrations were consistent with changes in fecal fermentation-related profiles associated with the high-fiber diet.

### Effects of a high-fiber diet on fecal microbiota in tibetan pigs

DF exerts beneficial effects on the gut largely by modulating the composition of the gut microbiota and its derived metabolites. As a complex and dynamic ecosystem, the gut microbiome plays a crucial role in host health, and the intricate interplay among diet, gut microbes, and the host has been extensively studied [56–59]. In light of this, we further investigated the specific impacts of a high-fiber diet on the intestinal health of Tibetan pigs. Generally, a high microbial α-diversity is considered a hallmark of a robust and healthy gut microbiome, whereas diminished diversity is often indicative of dysbiosis or potential disease states [60, 61]. Our results demonstrated that the high-fiber diet elevated α-diversity indices (Chao1, Faith’s_PD, observed species, and Shannon), suggesting that appropriate fiber inclusion may contribute to intestinal health in Tibetan pigs. Typically,. Firmicutes and Bacteroidetes are generally recognized as dominant phyla in the porcine gut [62]. This phylum-level pattern was also observed in fecal samples from both the CON and HF groups in the present study and is broadly consistent with previous reports in pigs. To further identify taxa potentially associated with dietary treatment, genus-level analyses were performed. The results identified *p-75-a5*, *SMB53*, *Coprococcus*, *Oscillospira*, *Lactobacillus*, and *Prevotella* as potential marker genera related to the high-fiber diet. Compared with the CON group, the HF group exhibited significant increases in the abundances of *p-75-a5* and *Coprococcus*. The genus *p-75-a5*, belonging to the family Erysipelotrichaceae, is strongly associated with protein and lipid digestion [63]. Its enrichment in the HF group likely reflects the increased availability of carbohydrate substrates provided by the high-fiber diet. This aligns with Jiao et al. [64], who reported a positive correlation between *p-75-a5* abundance and the activities of amylase, carboxymethyl cellulase, and xylanase in ileal contents. Furthermore, the elevated abundance of *p-75-a5* may drive important downstream metabolic effects, such as promoting SCFA production [65]. *Coprococcus* is a Gram-positive anaerobic bacterium within the family Ruminococcaceae (phylum Firmicutes) [66]. It plays a vital role in the porcine gut microbiota, particularly in butyrate production, thereby influencing host metabolism, immunity, and intestinal health [67]. As the primary energy source for colonocytes, butyrate is essential for supporting epithelial integrity and function [68]. Additionally, it exerts anti-inflammatory effects, modulates intestinal immune responses, and enhances barrier function by stimulating mucus production and tight junction protein expression. This collectively reduces intestinal permeability and protects against pathogen invasion [69].

Our comparative analysis showed that the HF diet was associated with higher relative abundances of *p-75-a5* and *Coprococcus*. These findings are consistent with the modulatory effects of dietary fiber on gut microbiota composition and suggest that these taxa may be related to changes in host metabolic and intestinal health-related indicators, as reported by Liu et al. [19]. Nevertheless, the potential interactions among these enriched taxa and the metabolic processes through which they may participate in dietary fiber fermentation remain to be clarified.

To further examine the associations among fecal microbiota, intestinal enzyme activities, and hindgut fermentation metabolites, Spearman’s rank correlation analysis was performed. The results showed that *p-75-a5* was positively correlated with cellulase activity, whereas *Coprococcus* was positively correlated with isobutyric acid. Isobutyric acid has been reported to support intestinal barrier function by reducing inflammation and oxidative stress, including downregulation of pro-inflammatory cytokine expression and enhancement of antioxidant capacity [70]. Therefore, the concurrent changes in *p-75-a5*, *Coprococcus*, cellulase activity, and isobutyric acid may reflect coordinated microbial and metabolic responses to the high-fiber diet. Together, these observations suggest that the enrichment of specific bacterial taxa is associated with improved fiber-utilization-related indicators in Tibetan pigs, although causal relationships require further validation.

Despite these findings, several limitations should be considered. The present post-weaning results complement our previous observations in growing Tibetan pigs fed high-fiber diets [20]. However, the microbiota–enzyme–SCFA associations observed in this study were mainly based on 16 S rRNA sequencing and correlation analysis; therefore, they should be interpreted as associations rather than evidence of causality. Functional validation, such as metagenomic analysis, metabolomic profiling, bacterial isolation, and in vitro fermentation assays, is required in future work to clarify whether these taxa participate in dietary fiber degradation and SCFA production. In addition, the reliance on fecal samples for SCFA and enzyme quantification may not fully reflect spatial differences in production, absorption, and microbial activity across different intestinal segments. Finally, the use of a single fiber inclusion level precludes the determination of an optimal fiber utilization threshold. Future studies incorporating fiber gradients, diverse fiber sources, and functional validation approaches may help further clarify the microbial and metabolic features associated with dietary fiber utilization in Tibetan pigs.

## Conclusions

In this study, a 14.94% NDF diet did not compromise the growth performance of post-weaning Tibetan piglets weighing 10–20 kg. This diet was associated with changes in the gut microenvironment, including higher relative abundances of *p-75-a5* and *Coprococcus*, increased fecal cellulase activity, and higher fecal concentrations of acetate, propionate, and butyrate. These findings suggest that this level of fiber inclusion may support favorable fiber-fermentation responses in Tibetan pigs. Overall, this study provides useful theoretical and experimental support for formulating cost-effective, high-fiber nursery diets for Tibetan pigs.

## Data Availability

The datasets generated and analyzed during the current study are available in the NCBI BioProject repository under accession number PRJNA1457488.
